# Religion and Animal Welfare—An Islamic Perspective

**DOI:** 10.3390/ani7020011

**Published:** 2017-02-17

**Authors:** Sira Abdul Rahman

**Affiliations:** Bangalore Veterinary College, 123, 7th B Main Road, 4th Block West, Jayanagar, Bangalore 560011, India; shireencva@gmail.com; Tel.: +91-80-2663-5210; Fax: +91-80-2663-5210

**Keywords:** Islam, animal welfare, halāl, slaughter

## Abstract

**Simple Summary:**

Cruelty to animals occurs during production, handling, transport, and slaughter in most countries where Islam is a major religion. Most of the people involved in this, such as those involved in the transport of animals, animal handlers, and butchers, are Muslims. However, many Muslims and Islamic religious leaders are not aware of this cruelty. Islam is a religion that shows compassion to animals as mentioned in the holy book Qur’an and sayings of the Prophet Mohammed (pbuh). This paper highlights what Islam says of the welfare of animals and how animal welfare can be improved by sensitizing all Muslims and religious leaders to the teachings on animal welfare in the Qur’an and the Hadiths so that they can influence their followers.

**Abstract:**

Islam is a comprehensive religion guiding the lives of its followers through sets of rules governing the personal, social, and public aspects through the verses of the Holy Qur’an and Hadiths, the compilation of the traditions of Prophet Mohammed (pbuh), the two main documents that serve as guidelines. Islam is explicit with regard to using animals for human purposes and there is a rich tradition of the Prophet Mohammad’s (pbuh) concern for animals to be found in the Hadith and Sunna. Islam has also laid down rules for humane slaughter. In many countries animals are killed without pre-stunning. Regardless of pre-stunning, such meat should not be treated as halāl or at least be considered as Makrooh (detestable or abominable), because the animals have been beaten or treated without compassion during production, handling, transport, and slaughter. Many Muslims and Islamic religious leaders are not aware of the cruelty that is routinely inflicted on animals during transport, pre-slaughter, and slaughter in many Islamic countries. There is an urgent need to sensitize all Muslims to the teachings of animal welfare in the Qur’an and the Hadiths. A campaign is needed to apprise religious leaders of the current cruelty that occurs during transport and slaughter.

## 1. Introduction

There is considerable debate on the role of religion in animal welfare, with implications for the study of welfare for welfare assessment and for implementation of solutions to welfare problems. In Islam, the law is a privileged means of access to the sacred. For most Muslims, Islamic normativity (fiqh or shari’a) is an essential part of being a Muslim. The demand for and production of authoritative rulings is one form of social expression of normative Islam. 

There are many published papers on how Islam provides an ethic of environmental concern and nonhuman animal protection [1,2,3,4,5,6,7,8,9,10,11,12,13,14,15]. Islamic law is most prescriptive in its insistence on humane treatment. The killing of nonhuman animals for meat and hides by halāl (that is, permissible based on a set of ethical and religious standards) methods is obligatory, with meat considered forbidden (Makrooh) if the nonhuman animal has in any way been subjected to inhumane treatment [3]. Regenstein [16] cites several sources that stipulate the need to ensure humane and efficient practices are fulfilled, including edicts from the second and fourth Caliphs. Generally, the killing of wildlife for any other reasons than food is always prohibited, as is the caging of birds, sports hunting, and animal baiting [8]. 

## 2. The Relevance of Animal Welfare under Islam

Islam provides considerable support for the importance of animal welfare. There is a rich tradition of the Prophet Mohammad’s (pbuh) concern for animals to be found in the Hadith and Sunna, and Islam provides considerable support for the importance of animal welfare.

The Qur’an [14] is explicit with regard to using animals for human purposes. A closer look at the teachings of the Qur’an and tradition reveals teachings of kindness and concern for animals.

For example:

- And cattle He has created for you (men); from them ye derive warmth and numerous benefits, and of their (meat) ye eat. **Surrah An-Nahl 16:5**
- And they carry your heavy loads to lands that ye could not (otherwise) reach except with souls distressed: for your Lord is indeed Most Kind, Most Merciful. **Surrah An-Nahl 16:7**
- And (He has created) horses, mules, and donkeys, for you to ride and as an adornment; And he has created other things of which ye have no knowledge. **Surrah An-Nahl 16:8**
- We have made animals subject to you, that ye may be grateful. **Surrah Al**
- **Haj 22:36**
- There is not a moving (living) creature on earth, nor a bird that flies with its two wings, but are communities like you. We have neglected nothing in the Book, then unto their Lord they (all) shall be gathered. **Surrah Al-Anam 6:38**
- Seest thou not that it is Allah Whose praise all beings in the heavens and on earth do celebrate, and the birds (of the air) with wings outspread? Each one knows its own (mode of) prayer and praise, and Allah knows well all that they do. **Surrah An-Noor 24:41**
- Qur’an actually forbids human actions which may lead to harm; transgress not in the balance, and weigh with justice, and skimp not in the balance … earth, He set it down for all beings **Surrah Ar-Rahman 55:8–10**

We now have a view of animals that shows them not merely as resources, but as creatures dependent on God (Allah). Animals are seen to have their own lives and purpose, valuable to themselves and to Allah above and beyond any material value they may provide to humanity.

The Qur’an is not the only Islamic source for messages of kindness towards animals.

There is a rich tradition of the Prophet Mohammed’s (pbuh) concern for animals to be found in the Hadith and Sunna. For example,

- The Prophet Muhammad (pbuh) condemned the beating of animals and forbade striking, branding, or marking them on the face.
- He cursed and chastised those who mistreated animals and gave praise to those who showed kindness;
- He also instituted radical changes against the practice of cutting off the tails and humps of living animals for food.

One Hadith quotes Prophet Muhammad (pbuh) as saying:

“A good deed done to an animal is as meritorious as a good deed done to a human being, while an act of cruelty to an animal is as bad as an act of cruelty to a human being.”

Prophet Muhammad (pbuh) was especially vocal in his disapproval of the cruel practices of notching and slitting of ears of animals and the practice of putting painful rings around the necks of camels. (Hadith: Bukhari)

Below are just a few well-known examples from the hadith (traditions):

- “There is a reward (ajr) for helping any living creature.” (Hadith: Bukhari and Muslim)
- “It is a great sin for man to imprison those animals which are in his power.” (Hadith: Muslim)
- “The worst of shepherds is the ungentle, who causes the beasts to crush or bruise one another.” (Hadith: Muslim)
- “You will not have secure faith until you love one another and have mercy on those who live upon the earth.” (Hadiths: Bukhari, Muslim, and Abu Dawud)
- “Fear God in these mute animals, and ride them when they are fit to be ridden, and let them go free when … they (need to) rest.” (Hadith: Abu Dawud)
- “There is no man who kills a sparrow or anything beyond that, without its deserving it, but God will ask him about it.” (Hadiths: Ahmad and al-Nasai)
- The grievous things are: shirk (polytheism); disobedience to parents; the killing of breathing beings …” (Hadiths: Bukhari and Muslim)
- “May god curse anyone who maims animals.” (Hadith: Bukhari)
- “Whoever is kind to the creatures of God is kind to himself.” (Hadith: Bukhari)
- “There is none amongst the Muslims, who plants a tree or sows seeds, and then a bird, or a person or an animals eats from it, but is regarded as a charitable gift for him”(Hadith: Bukhari)

These examples clearly indicate how Islam treats any animal with kindness.

## 3. Islam and Rules Concerning the Slaughter of Animals

The humane slaughter of animals is strongly supported in the Islamic tradition. For example, Sahih Muslim (Book 21, Chapter 11, Number 4810) records Prophet Mohammad *(*pbuh*)* saying:

“Verily Allah has enjoined goodness to everything; so when you kill, kill in a good way and when you slaughter, slaughter in a good way. So every one of you should sharpen his knife, and let the slaughtered animal die comfortably.”

Prophet Muhammad (pbuh) has also said, “When one of you slaughters, let him complete it”, meaning that one should sharpen the knife well and feed, water, and soothe the animal before killing it.

He also said, “Do you intend (on) inflicting death on the animal twice—once by sharpening the knife within its sight, and once by cutting its throat?”

Islam has also laid down Other Rules for humane slaughter as indicated by a combination of Hadiths, including the following:

1. Animals should have a preslaughter rest, and be well fed and well looked after at the point of slaughter.
2. The animals must be alive or deemed to be alive at the time of slaughter.
3. Slaughter must be performed by a Muslim (who is of sound mind, mature, and fully understands the Islamic procedure and conditions for slaughtering of animals).
4. that are slaughtered should be securely restrained, particularly the head and neck, before cutting the throat.
5. Operator competence is of great importance in order to carry out satisfactory halāl slaughter.
6. tools and other implements used must be for the slaughter of halāl animals only.
7. The knife must be razor sharp and without blemishes and damage. For animals with normal necks, the act of slaughter must begin with an incision on the animal’s neck just before the glottis, and for animals with long necks such as chicken, turkeys, ostriches, camels, etc., the incision must be before the glottis.
8. The animal’s trachea and esophagus must be severed. The spinal cord should not be cut and the head not severed completely so as to induce immediate and massive hemorrhage. In certain mazhab (school of thought), uttering the phrase “bismillah” immediately before the slaughter is compulsory. In others, such utterance is highly encouraged.
9. must be done once only. The slaughtering implement must not be lifted off the animal during slaughtering. Any lifting is construed as one act of slaughter. Multiple acts of slaughter on one animal are prohibited.
10. Slaughter the animal in such a way that its life departs quickly, and it is not left to suffer.
11. must be spontaneous and complete.
12. should not be shackled and hoisted before bleeding.
13. should be done only after the animal has lost consciousness. Restraining equipment should be comfortable for the animal.
14. Further preparation and dressing of the carcass must be delayed until all signs of life and cerebral reflex have disappeared.

Shackling and hoisting conscious animals seems to violate both the humane intent of Islamic slaughter law, and Prophet Muhammad’s (pbuh) comments on the process of slaughter.

Regarding stunning, Al-Masri [3] notes that stunning has been declared as acceptable by a fatwa (unanimous verdict) of the Al-Azhar University in Cairo. Furthermore, the Muslim World League declared in 1986 that pre-slaughter stunning is lawful when the weakest electric current renders a nonhuman animal unconscious before slaughter [3]. Requirements and methods of stunning which are acceptable by Islamic authorities in countries such as Malaysia have been published [17].

Eating meat produced using cruel methods violates the Prophet Muhammad’s (pbuh) general precept to cause animals no pain before their slaughter, as well as more specific injunctions regarding the treatment of food animals. Indeed, if animals have been subjected to cruelty in transport and slaughter, or to general cruelty, meat from them is considered by Islam as impure and unlawful to eat (Makrooh). The flesh of animals killed by cruel methods (Al-Muthiah) is carrion (Al-Mujaththamah). Even if these animals have been slaughtered in the strictest Islamic manner, if cruelties were otherwise inflicted on them, their flesh is still forbidden (Haram) food.

“Oh, ye messengers! Eat of the good things {tayyibat} and do righteous deeds. Surely, I know what you do” (**Qur’an 23:51**).

“Oh believers! Eat what We have provided for you of lawful and good things, and give thanks for Allah's favour, if it is He whom you serve” (**Qur’an 2:172; 16:114**).

The word “Tayyib”, translated as “good”, “pure”, or “wholesome”, means “pure”, both in the physical and the moral sense. 

In summary, the main counsel of Islam for the slaughter of animals for food is to do it in the least painful manner. All the Islamic laws on the treatment of animals, including the method of slaughter, are based on compassion, fellow-feeling, and benevolence. 

## 4. What is Prevalent Today?

Many current practices are not in accordance with the above teachings and may result in great cruelty to animals. Handling of animals before and during transport is often cruel. Some animals are marched on foot for several days. During such transport, animals may lose weight and may be beaten unnecessarily. Many animals are not fed and watered en route. Animals—young and old, big or small—may be tied in twos and fours in order to reduce the number of animal minders or personnel on the trail. Such tying results in injury and fatigue to the animals. Some animals are beaten and forced to move quickly in order to reach markets and abattoirs on time. Those that fall down may be whipped to force them to rise [1]. 

Similarly, needless suffering is inflicted on animals that are transported three or four days together in overcrowded, ill-ventilated, trucks, especially in hot, humid weather.

Harsh conditions also occur at slaughter plants. Animals may be held in primitive facilities without shade, and animals may be restrained by short tethers. At the point of slaughter, animals are often struck and beaten to make them enter the slaughter facilities.

## 5. What Needs to be Done?

Many Muslims and Islamic religious leaders are not aware of the cruelty that is routinely inflicted on animals during transport, at pre-slaughter, and at slaughter in many Islamic countries. There is an urgent need to sensitise all Muslims to the teachings on animal welfare in the Qur’an and the Hadiths. This approach is bound to be effective in influencing the majority of Muslims in the livestock trade, especially the slaughter man in treating animals more humanely. This needs to be done by intervention at the highest level by religious bodies and organisations, which could be most effective in giving rulings (fatwas) on this issue [1]. Poor practices and animal welfare abuses occurring during halāl meat production has been reviewed [18], with ways and means suggested to improve animal welfare especially using Mosque-based sermons by Imams to increase awareness of animal welfare issues. The Dialrel project [19] reviewed current practices during halāl and Sechita slaughter in cattle, sheep, goat, and poultry in Belgium, Germany, Italy, the Netherlands, Spain, UK, Turkey, and Australia, and the report discussed various stakeholders including Muslim and Jewish representatives.

Progress might be achieved by taking the following measures.

1. A campaign is needed to apprise religious leaders of the current cruelty that occurs during transport and slaughter, for example by slides and videos. This should be done by competent and knowledgeable individuals who are also aware of the Islamic principles of animal welfare, preferably by Muslims in order to give authenticity to their claims.
2. The creation of animal welfare legislation, including animal transport and slaughter, according to the World Organisation for Animal Health (OIE) standards and Islamic principles.
3. Government officials in charge of livestock, especially at abattoirs, should be sensitised to the concepts of animal welfare and how these relate to Islamic principles.
4. Abattoirs should be equipped with the facilities required for the good application of animal welfare standards, including unloading facilities, slaughtering boxes, and well-trained personnel to implement correct halāl slaughter.
5. The OIE animal welfare standards, especially those dealing with land transport and slaughter of animals for human consumption, which were adopted in 2005 by OIE Members, need to be more strictly implemented by governments.

The OIE encourages Veterinary Services to enter into dialogue with religious authorities with the objective of raising awareness of the importance of animal welfare and reducing animal suffering globally. In order to achieve this, the OIE would, if required, assist member countries by providing expertise of Muslims knowledgeable both in Islamic Shariah and Animal Welfare.

## 6. Conclusions

Since cruelty to animals occurs during production, handling, transport, and slaughter in most countries where Islam is a major religion, Muslims and Islamic religious leaders need to be sensitized to this issue with reference to the teachings of animal welfare in the Qur’an and the Hadiths. To achieve this objective a campaign is needed with the help of animal welfare organizations and the World Organization for Animal Health (OIE). This will greatly influence the majority of Muslims in the livestock trade in treating animals more humanely.
